# Global Proteomics Analysis of the Response to Starvation in C. elegans*[Fn FN2]

**DOI:** 10.1074/mcp.M114.044289

**Published:** 2015-05-11

**Authors:** Mark Larance, Ehsan Pourkarimi, Bin Wang, Alejandro Brenes Murillo, Robert Kent, Angus I. Lamond, Anton Gartner

**Affiliations:** From the ‡Centre for Gene Regulation and Expression, College of Life Sciences, University of Dundee, Dow St, Dundee, United Kingdom, DD15EH

## Abstract

Periodic starvation of animals induces large shifts in metabolism but may also influence many other cellular systems and can lead to adaption to prolonged starvation conditions. To date, there is limited understanding of how starvation affects gene expression, particularly at the protein level. Here, we have used mass-spectrometry-based quantitative proteomics to identify global changes in the *Caenorhabditis elegans* proteome due to acute starvation of young adult animals. Measuring changes in the abundance of over 5,000 proteins, we show that acute starvation rapidly alters the levels of hundreds of proteins, many involved in central metabolic pathways, highlighting key regulatory responses. Surprisingly, we also detect changes in the abundance of chromatin-associated proteins, including specific linker histones, histone variants, and histone posttranslational modifications associated with the epigenetic control of gene expression. To maximize community access to these data, they are presented in an online searchable database, the Encyclopedia of Proteome Dynamics (http://www.peptracker.com/epd/).

The irregular availability of food provides a major challenge for organismal survival. Animals have to compromise between growth and survival in response to limited food sources. The nematode *C. elegans* provides an excellent model system to study the conserved regulatory circuits that link nutrient availability, starvation response, and longevity. In its natural environment *C. elegans* can experience conditions of both feast and famine (1). Within the 3 to 4 day life cycle of the nematode, embryogenesis is supported by maternally provided nutrients and occurs in the absence of food. Larval development, however, requires feeding and progresses through four developmental stages (L1-L4). The effect of starvation varies at these different developmental stages. Hatching of L1 stage larvae in the absence of food leads to a starvation response without overt morphogenesis that allows for ∼2 weeks of survival (2). Mid- and late-stage L4 larvae, upon starvation, develop into adults but preserve resources by reducing germ cell proliferation and thereby holding a limited number of embryos (3, 4).

Conserved genetic pathways have been identified that link nutrient availability to metabolic remodeling, stress resistance pathways, and enhanced life span. Many of these pathways overlap and include TOR[Fn G1], AMPK, autophagy, and insulin/IGF-1 signaling. PHA-4 is a FOXA transcription factor downstream of TOR kinase that is inactivated in the fed state (5). Upon starvation, inhibition of TOR leads to de-repression of PHA-4 activity and the regulation of transcription important for starvation stress survival (5). Insulin and insulin-like signaling also plays a role in starvation responses and converges on the DAF-16 FOXO transcription factor, which in the absence of food activates stress resistance and related longevity pathways (6, 7). Other pathways, including the AMP kinases (8) and AP1 (Jun/Fos)-dependent transcriptional control are also linked to metabolic remodeling and longevity (9–12). Also, DAF-16 and the nematode Rb homolog (LIN-35) globally affect transcription upon starvation at the L1 developmental stage (13). However, the effect of starvation on chromatin remodeling has not been examined previously.

Quantitative, proteome-wide changes in response to acute starvation have not previously been documented in detail in any organism. Using *Drosophila,* larval fat bodies were studied in response to amino acid starvation (14). Another study analyzing ∼700 proteins reported changes occurring in the fly hemolymph, showing that yolk, storage, and fat body proteins are down-regulated in response to starvation (15). A study on rodents focused on ∼200 selected proteins involved in metabolism and insulin signaling that were extracted from mouse livers. This analysis of livers derived from mice that were fed on either a normal or high fat diet and both then compared in response to starvation revealed differences in protein levels that were dependent on the genetic background of the mouse strains analyzed (16).

We have used the stable isotope labeling with amino acids in cell culture (SILAC) approach for quantitative mass-spectrometry-based proteomics (17) and improved on the efficiency of previous nematode SILAC studies (18, 19). The focus of this study is on changes in protein abundance, which is particularly important when the major regulatory mechanisms involved are unknown (20–22). We have characterized specific sets of proteins whose abundance levels are either up- or down-regulated in response to starvation, thereby identifying metabolic and signaling pathways responding to starvation stress. Interestingly, we also detect specific histone variants and posttranslational modifications that are regulated in response to starvation.

## EXPERIMENTAL PROCEDURES

### 

#### 

##### Materials

The EZQ protein assay and CBQCA assay were from Thermo Fisher Scientific (Waltham, MA). Triscarboxyethylphosphine (TCEP) (bond-breaker neutral pH solution) was from Pierce (Thermo Fisher Scientific). Trypsin MS-Grade was from Promega. Sep-Pak tC18 μ-elution 96-well plates were from Waters. The Pepmap C18 (2 cm × 75 μm) trap columns and EasySpray C18 columns (2 μm particles, 50 cm × 75 μm) were from Thermo Fisher Scientific. Complete protease inhibitor mixture tablets and PhosStop phosphatase inhibitor tablets were from Roche. All other materials were obtained from Sigma.

##### C. elegans Strains, Maintenance, and Starvation

*C. elegans* N2 Bristol strain was used and maintained at 20 °C unless otherwise indicated. The *his-71::gfp* strain was obtained from the CGC. For isotopic labeling, *C. elegans* were grown on NGM-N plates as described previously (18). *C. elegans* were monitored every day and L4 larvae stage *C. elegans* of the following generation (F1) were picked onto a fresh plate (plate 2). This procedure was followed unless indicated otherwise.

##### C. elegans SILAC

The SLE1 auxotrophic derivative of *Escherichia coli* HT115, containing an orn-1 RNAi feeding construct, was used as previously described (18), with genotype argA, lysA, F-, mcrA, mcrB, IN(rrnD-rrnE)1, lambda -, rnc14::Tn10 (DE3 lysogen: lavUV5 promoter -T7 polymerase).

##### Lifespan Assay

A lifespan assay was performed at 20 °C as previously described (23).

##### L1 Starvation Assay

An L1 starvation assay was performed as described previously, using a 5-day starvation stress and a 50 h recovery time prior to counting (24).

##### SDS-PAGE and Immunoblotting

SDS-PAGE and immunoblotting were performed as described previously (25).

##### M9 Minimal Media

M9 minimal media (Na_2_HPO_4_ 5.8 g L^−1^, KH_2_PO_4_ 3 g L^−1^, NaCl 0.5 g L^−1^, NH_4_Cl_2_ 1 g L^−1^, glucose 0.2% (w/v), MgSO_4_ 1 mm, thiamine 0.01% (w/v)) was prepared by mixing 100 ml of 10 x M9 salts (420 mm disodium phosphate, 240 mm monopotassium phosphate, 90 mm sodium chloride, 190 mm ammonium chloride) and 893 ml of deionized water, and autoclaved (120 °C for 20 min). After cooling to 55 °C, 5 ml of 40% (w/v) glucose, 1 ml of 1 m MgSO_4_ and 1 ml of 1% (w/v) thiamine were added. Lysine and arginine were added to 40 μg ml^−1^ final concentrations from either a 73 mg ml^−1^ or 42 mg ml^−1^ stock (in PBS), respectively. A single bacterial colony freshly streaked on an LB plate from a frozen stock was used to start a 200 ml culture. Bacteria were incubated at 37 °C under agitation (220 rpm) until OD_600 nm_ = 1 was reached. Bacteria were concentrated by centrifugation (8,000 g for 20 min) to OD_600 nm_ = 50 and 5 ml plated onto NGM-N plates. Plates were then stored at 20 °C and used within 7 days.

##### RNAi Feeding

RNA interference experiments were performed according to the feeding method with the following modifications: Cells carrying the corresponding feeding vector were grown in 2 ml of M9 minimum media supplemented with either heavy or light arginine and lysine (40 μg ml^−1^), and carbenicillin (1 μg ml^−1^) at 37 °C until OD_600 nm_ = 1. Double-stranded RNA expression was induced by the addition of IPTG (1 mm final concentration) for 3 h. Bacteria were pelleted and resuspended in 1 ml of worm M9 buffer supplemented with IPTG 1 mm, carbenicillin 1 μg ml^−1^ to which ∼500 L1 larvae stage worms were added. This mixture was transferred into a 50 ml falcon tube to allow oxygenation and incubated at 25 °C overnight under gentle agitation (150 rpm). Worms and bacteria were spun down the following day, plated on 9 cm NGM-N plates, and incubated at 20 °C. The following worm generation (F1) was analyzed for the corresponding phenotype.

##### Labeling C. elegans Plates

*C. elegans* were grown on NGM plates that do not contain any nitrogen source (called NGM-N plates). For 1 L of NGM-N medium, 3 g of NaCl and 12 g of agarose (Invitrogen, molecular grade) were mixed with 970 ml of deionized water and autoclaved (120 °C for 20 min). After cooling down to 55 °C, the following compounds were added: 1 ml of 1 m CaCl_2_, 1 ml of 1 m MgSO_4_, 25 ml of 1 m KPO_4_, 1 ml of cholesterol 5 mg ml^−1^ (in ethanol), and 1 ml of nystatin (10,000 units ml^−1^). 9 cm plates were used.

##### Starvation Time Course and Wild-Type versus Mutant Nematode Proteome Analysis

##### Generation of C. elegans Total Nematode Lysates

*C. elegans* were collected from plates by flushing with PBS and washed three times with PBS. *C. elegans* were pelleted and resuspended in ice cold 100 μl PBS containing complete protease inhibitors, PhosStop, and 5 mm N-ethylmaleimide prior to snap-freezing in liquid nitrogen. For lysis, *C. elegans* were thawed on ice and sodium dodecyl sulfate (SDS) was added to generate a 2% (w/v) final concentration. TCEP (reducing agent) was added to a final concentration of 25 mm; the solution was vortexed and then heated to 65 °C for 10 min. N-ethylmaleimide was added to 50 mm final concentration for 30 min at room temperature for alkylation of cysteine residues. Zirconia beads (0.7 mm, Biospec Products) were added to make a ∼50% (v/v) slurry, and the sample was bead-beaten (Mini Beadbeater 8, Biospec Products) for 1 min at room temperature. The lysate was then centrifuged for 10 min at 17,000 g at room temperature. A BCA assay (Pierce, Rockford, IL) was performed on the supernatant and for SILAC mixing; equal proportions of protein were combined.

##### Offline Peptide Strong Anion Exchange (SAX) Chromatography

Lysates were chloroform-methanol precipitated (26) and resuspended in 8 m urea, 100 mm Tris-HCl pH 8.0, 1 mm CaCl_2_. After dilution to 1 m urea with 100 mm Tris-HCl, pH 8.0, 1 mm CaCl_2,_ the lysate was digested with trypsin at an enzyme:protein ratio of 1:50 (Promega). The peptides were then desalted 500 mg SepPak tC18 cartridges (Waters), dried in a centrifugal evaporator and resuspended in 50 mm borate, pH 9.3. NaOH was added to ensure pH was above pH 9.0 in samples prior to separation. Peptides were separated with an AS24 strong anion exchange column (Thermo Fisher Scientific) using an Ultimate 3000 UHPLC (Thermo Fisher Scientific) as described previously, with some modifications (27). Briefly, buffer A was 10 mm sodium borate-NaOH, pH 9.3, and buffer B was 10 mm sodium borate-NaOH, pH 9.3, 0.5 m sodium chloride. Peptides were eluted using an exponential gradient into 16 × 560 μl fractions. The peptide fractions were desalted using Sep-Pak 10 mg tC18 μ-Elution 96-well plates (Waters) and then resuspended in 5% formic acid for LC-MS/MS analysis.

##### Starvation 16 h Biological Triplicate Proteome Analysis

##### Subcellular Fractionation of C. elegans

The QProteome Cell Compartment fractionation kit (Qiagen) was used to fractionate worms according to the manufacturer's tissue fractionation protocol. Briefly, *C. elegans* were collected by flushing with PBS and washed three times with PBS. Freshly harvested *C. elegans* were pelleted and resuspended in ice cold buffer 1 containing complete protease inhibitors. Zirconia beads (0.7 mm, Biospec Products) were added to make a ∼50% (v/v) slurry and the sample was bead-beaten (Mini Beadbeater 8, Biospec Products) for 5 s at 4 °C, and lysis was checked by microscopy. The lysate was then processed for the remaining steps as per the manufacturer's instructions. A BCA assay (Thermo Fisher Scientific) was performed on each fraction prior to denaturing gel filtration chromatography of each.

##### Denaturing Gel Filtration Chromatography, Trypsin Digestion, and Peptide Clean-up

Using a Dionex Ultimate 3000 HPLC system (Thermo Fisher Scientific), subcellular fractions in 6 m guanidine-HCl were injected (20 μl per injection—80 μg protein) onto a mAbPacSEC column (Dionex) equilibrated with 6 m urea, 2 m thiourea, and 0.1 m Tris-HCl, pH 7.0. The flow rate was 0.2 ml min^−1^ and 16 × 100 μl fractions were collected using a low protein binding 96-deep well plate (Eppendorf). Trypsin digestions were performed at pH 8.0 by adding three volumes of 0.1 m Tris-HCl, pH 8.0, 1 mm CaCl_2_ to each fraction to dilute the urea. 500 ng of trypsin were subsequently added to each well. The plate was sealed with a rubber mat, vortexed, and incubated overnight at 37 °C. Trifluoroacetic acid was added to 1% (v/v) final concentration, and peptides were purified using an Oasis HLB 96-well μ-elution plate. Peptides were eluted in 100 μl of 50% (v/v) acetonitrile and dried in a centrifugal evaporator prior to resuspension in 5% (v/v) formic acid. Peptide concentrations were determined using the CBQCA assay (Invitrogen) after 25-fold dilution of peptide samples in 10 mm sodium borate-NaOH, pH 9.3.

##### LC-MS/MS and Analysis of Spectra

Using a Thermo Fisher Scientific Ultimate 3000 RSLCnano UHPLC, peptides in 5% (v/v) formic acid (final volume ∼10 μl) were injected onto an Acclaim PepMap C18 nano-trap column. After washing with 2% (v/v) acetonitrile and 0.1% (v/v) formic acid, peptides were resolved on a 50 cm × 75 μm C18 EasySpray reverse phase analytical column with integrated emitter over a gradient from 2% acetonitrile to 35% acetonitrile over 140 min with a flow rate of 200 nl min^−1^. The peptides were ionized by electrospray ionization at +2.0 kV. Tandem mass spectrometry analysis was carried out on a Q-Exactive mass spectrometer (Thermo Fisher Scientific) using HCD fragmentation. The data-dependent acquisition method used acquired MS/MS spectra on the top 10 most abundant ions at any one point during the gradient. All of the RAW MS data have been deposited to the ProteomeXchange Consortium (http://proteomecentral.proteomexchange.org) via the PRIDE partner repository with the dataset identifier PXD001723. The RAW data produced by the mass spectrometer were analyzed using the quantitative proteomics software MaxQuant including the Andromeda search engine (28) (http://www.maxquant.org, version 1.3.0.5). Peptide- and protein-level identification were both set to a false discovery rate of 1% using a target-decoy-based strategy. The databases supplied to the search engine for peptide identifications were both the *C. elegans* and *E. coli* Uniprot databases (24/10/13) containing 26,147 and 4,311 entries, respectively. The mass tolerance was set to 7 ppm for precursor ions and MS/MS mass tolerance was set at 20 ppm. Enzyme was set to trypsin with up to two missed cleavages. Deamidation of Asn and Gln, oxidation of Met, pyro-Glu (with N-term Gln), and acetylation of the protein N terminus were set as variable modifications. N-ethylmaleimide on Cys was searched as a fixed modification. The output from MaxQuant provided peptide-level data as well as protein-level data, grouped by protein isoforms. We define a protein group as a set of unique peptides that are either shared between multiple protein isoforms or belong to a single protein isoform. While this may lead to higher redundancy, it enables us to quantify changes in individual protein isoforms.

##### Data Analysis

The R language (version 2.15.1) was used for data analysis. For all experiments peptide-level data were aggregated to generate data for each protein group, based on a set of unique peptides that were either shared between multiple protein isoforms or belonging to a single protein isoform. For the analysis of the starvation time course and the starvation response of wild-type *versus* the *his-71* null mutant, the mean and standard error of the mean were calculated from the SILAC ratios of each peptide corresponding to each protein group under each condition. For the three biological replicates starved for 16 h, the median, standard deviation and *p* value based on a Student's *t* test (unpaired, two-tailed, unequal variance) were calculated from the SILAC ratios of each protein group under each condition and negative control protein PRO-1 that did not change after starvation. For clustering analysis, the SILAC ratios were normalized to a mean log_2_ value of 0 and a standard deviation of 1. Hierarchical clustering was based on Euclidean distance measurement and a “complete” agglomeration method was used. The output of the clustering analysis has been presented as a heatmap, using the RColorBrewer library. The gene ontology analysis was carried out using the DAVID Functional Annotation Tool (http://david.abcc.ncifcrf.gov/) for biological processes (29). The full *C. elegans* proteome, supplied by DAVID, was selected as a background list. These data were then plotted to reduce redundancy using the REVIGO suite (30), using the default parameters.

##### qRT-PCR

Three biological replicates with three technical replicates each were used. Adult nematodes were picked from plates (10 per plate) into 100 μl of M9 salt buffer in screw-cap 1.5 ml tubes at 20 °C. After the animals settled to the bottom the majority of liquid was removed. 500 μl of Trizol (Life Technologies) was added and the samples immediately frozen at −80 °C. After thawing at room temperature, ∼100 μl of 0.7 mm zirconia beads (Biospec) were added and the samples were bead-beaten (Biospec) for 2 min at maximum speed at room temperature. Chloroform (100 μl per tube) was then added and samples shaken by hand for 15 s prior to centrifugation at 12,000 g for 15 min at 4 °C. The 200 μl of the upper phase was moved to a snap-cap 1.5 ml tube, and 200 μl of 100% ethanol added and vortexed. This was cleaned using an RNeasy mini kit (Qiagen) by applying the upper-phase ethanol mixture as the “sample” and following the manufacturer's instructions for subsequent steps. After elution in 50 μl of water the RNA content and purity was determined using a nanodrop (Thermo Fisher Scientific) by absorbance at 260 nm. 5 ng was used per qRT-PCR reaction on a 96-well Roche Light Cycler II. A one-step Quantifast SYBR Green RT-PCR kit (Qiagen) was used (20 μl reaction volumes per well) as per manufacturer's instructions. Primers used were *tbg-1* F: TCAACTGCTTCTCGGGTTCG, *tbg-1* R: TTGCGAAATCAACGTTTTGCTC, *gpd-2* F: GTACGACTCCACCCACGGA, *gpd-2* R: TTGTAGACCTTGATCTTGTGCTGCG, *acs-3* F: GCGAAAAAGACCAAGGCTCG, *acs-3* R: CCCCACCAGAAGTGAGAACC, *lys-4* F: AGAGCAGCTGGCCTCACCG, *lys-4* R: TCCTGCACTCTTCAAAGCATCAAG, *daf-16* F: TTGCCAAAGCATTGGAATCG, *daf-16* R: GGATCGAGTTCTTCCATCCG. The qRT-PCR program was as follows: 1) 50 °C 10 min; 2) 95 °C 5 min; 3) 95 °C 10 s; and 4) 60 °C 30 s (loop back to step 3 for 50 cycles). The *tbg-1* data were used for normalization across all samples.

## RESULTS

### 

#### 

##### Response of the C. elegans Proteome to Acute Starvation Stress

**Fig. 1.**
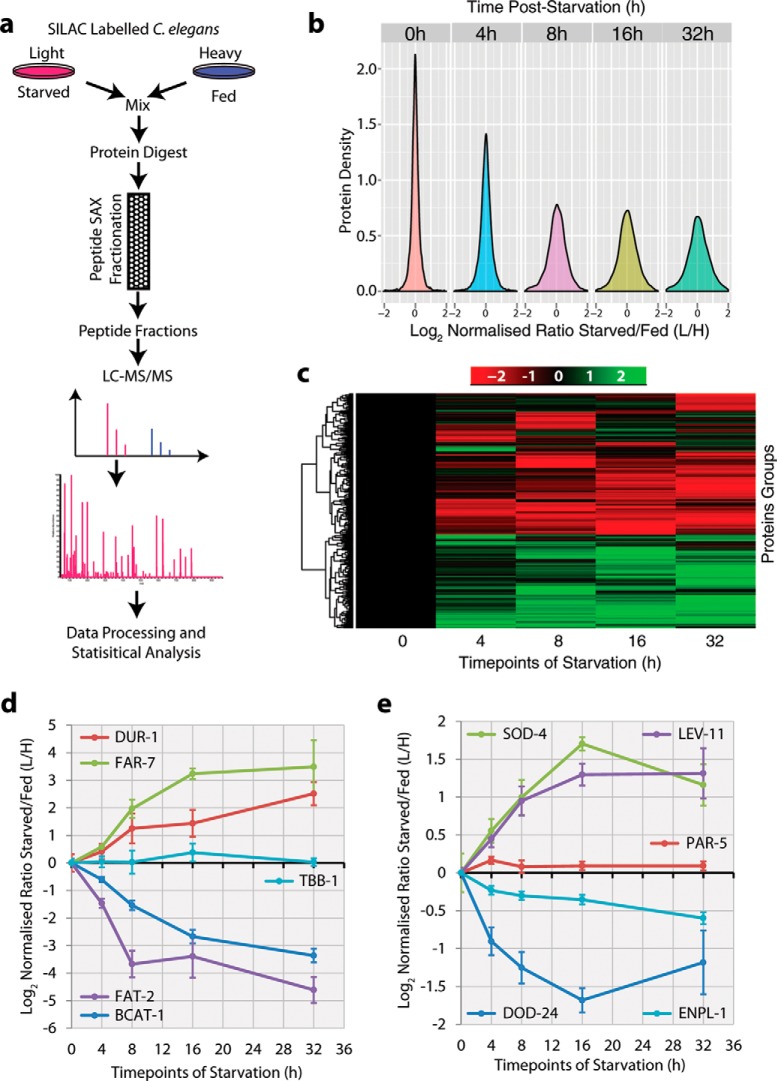
**Comprehensive proteome analysis of *C. elegans* after starvation stress.** (*A*) Workflow for SILAC-based quantitative mass spectrometry to identify starvation regulated proteins in *C. elegans*. This workflow was performed once for each time point of starvation stress. (*B*) Protein density histograms showing the response to starvation of ∼5,000 protein groups per time point after the time periods indicated. The mean SILAC ratio L/H (starved/fed) is shown separately for each time point on the *x* axis, with the same scale used for each graph. The protein density is indicated on the *y* axis. The colors represent each time point indicated at the top of each graph. (*C*) The profiles of proteins changing by more than twofold at any time point were hierarchically clustered. Decreased abundance is indicated by red shading, increased abundance by green shading. Each protein group (one per line) is shown along the *y* axis and the time after starvation stress is shown on the *x* axis. (*D*) Line graphs showing the mean peptide SILAC ratio *versus* time after starvation stress, for either selected proteins known to respond to starvation stress, or the negative control protein TBB-1 (Tubulin beta). Error bars show the standard error of the mean for the peptide SILAC ratio. (*E*) Line graphs showing the mean peptide SILAC ratio *versus* time after starvation stress, for selected proteins known to be regulated by either the transcription factor PHA-4 (FOXA, downstream of TOR) or DAF-16 (FOXO downstream of insulin/IGF-1 signaling). PAR-5 (14–3-3) served as a negative control. Error bars show the standard error of the mean for the peptide SILAC ratio.

To provide a comprehensive overview of the response of the *C. elegans* proteome to starvation, we performed a time course of food deprivation with SILAC labeled nematodes. SILAC “light” isotope-labeled nematodes were deprived of food (*E. coli*) for either 4, 8, 16, or 32 h, whereas “heavy” isotope-labeled nematodes were kept in the presence of food for the same periods (Fig. 1*A*). Starvation was initiated using a developmentally synchronized population of nematodes in the late L4 larval stage. The late L4 larval stage has previously been shown to result in the least perturbation upon prolonged starvation, assessed after more than 24 h of food removal (4). Equal amounts of protein (100 μg) derived from SILAC-labeled nematodes that were either fed or starved for the same time over the 32 h time course were mixed and processed for MS analysis (Fig. 1*A*). Mixed protein lysates were digested with trypsin, and the resulting peptides fractionated using SAX chromatography (27). Each fraction was subsequently analyzed by LC-MS/MS and the abundance of light- *versus* heavy-labeled peptides was quantified (Fig. 1*A*).

Peptides were identified and quantified using the MaxQuant software suite (28). From an average of over 45,000 unique peptides analyzed across the entire time course, more than 5,500 protein groups were quantified that were detected across all extracts from both the fed and starved nematodes and across all time points (Supplemental Table 1 for protein-level data and Supplemental Table 2 for peptide-level data; see methods). The classification of protein groups used here allows for the quantitative discrimination between related protein isoforms (for example, generated either by alternative splicing or representing closely related paralogues). On average, each protein group at each time point was defined by seven unique peptides. To ensure a high-quality dataset, we used a false discovery rate of less than 1% for both peptide and protein identification. In summary, this study identifies and quantifies the *C. elegans* proteome in-depth across the time course with high confidence.

To have an overview of the response of the proteome to starvation, histograms were generated showing the distribution across each time point of the log fold changes in protein abundance levels related to the number of proteins detected, represented by their density along the *x* axis (Fig. 1*B*). This shows that starvation leads to major changes in the *C. elegans* proteome, with both the number of proteins responding (either increasing or decreasing) and the degree of changes in abundance levels, progressively increasing over time post starvation onset until a plateau is reached at ∼8 h. We employed hierarchical clustering to characterize the changes in the proteome induced by starvation, using an inclusion threshold of at least a twofold change in abundance occurring in at least one time point (Fig. 1*C*, see methods). This identified >500 proteins that respond to starvation by either increasing or decreasing in abundance at some point during the time course.

Most proteins reached their maximum response within 16 h of starvation. Changes in abundance tended to occur progressively, albeit with the rate of change varying between different sets of proteins. For example, the abundance of the fatty acyl desaturase (FAT-2) was ∼16-fold down-regulated 8 h post onset of starvation and then remained relatively stable at this lower level for the remainder of the time course (Fig. 1*D*). In contrast, the branched chain amino acid aminotransferase (BCAT-1) gradually declined in abundance over the entire time course (Fig. 1*D*). Likewise, the fatty-acid-binding protein FAR-7 and the DUR-1 protein (dauer-associated protein), are both up-regulated. DUR-1 peaked in abundance within 8 h, while FAR-7 did not reach its maximum abundance until 16 h post starvation onset. In contrast, beta-tubulin (TBB-1) did not change in abundance throughout the time course of starvation.

A focused analysis of known PHA-4 (31) and DAF-16 targets (32), confirms that this study detects the expected regulation of these proteins in response to food deprivation (Fig. 1*E*). Thus, the protein abundance of the HSP90-homologue ENPL-1 (PHA-4 regulated) and the protein of unknown function DOD-24 (DAF-16 regulated), were decreased in abundance after starvation. In contrast, the proteins superoxide dismutase 4 (SOD-4, DAF-16 regulated) and tropomyosin (LEV-11, PHA-4 regulated) were both increased in abundance (Fig. 1*E*).

**Fig. 2.**
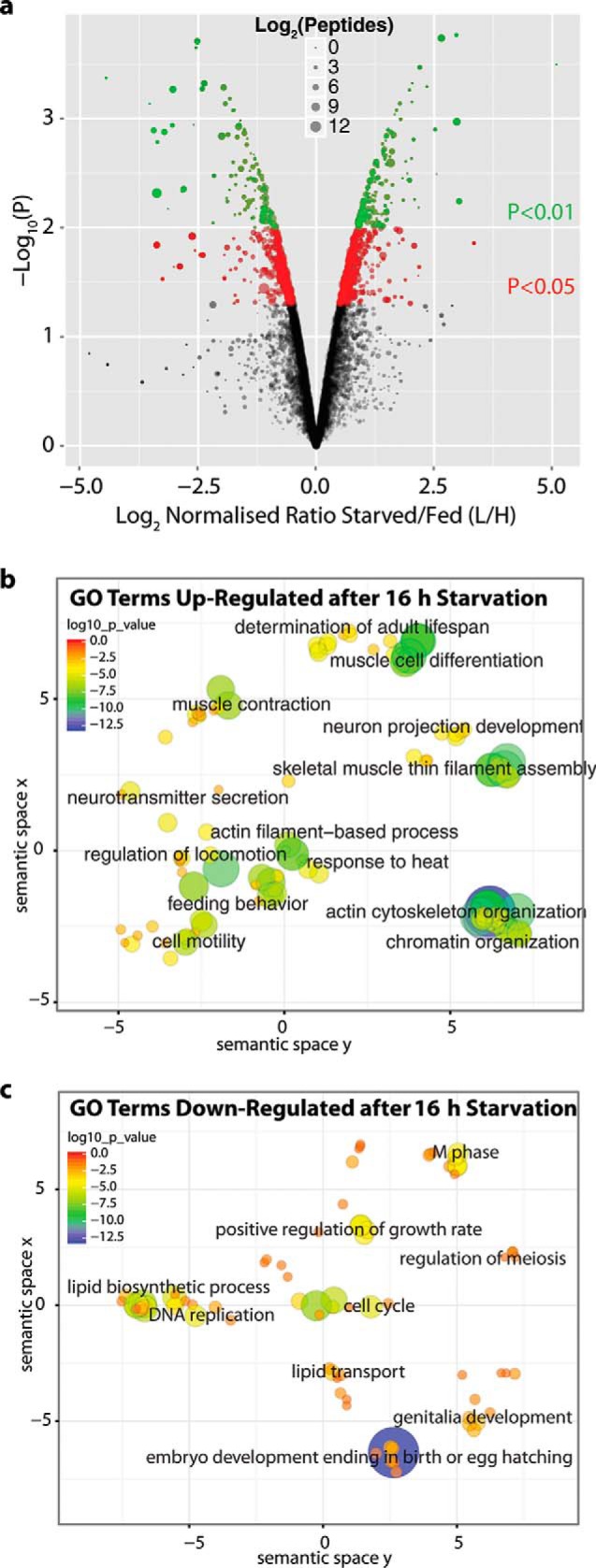
**Focused analysis of *C.* elegans after 16 h of starvation stress.** (*A*) The starvation stress response of ∼5,000 proteins is indicated with the median ratio of each protein group, represented by a spot (*n* = 3). The *y* axis shows a *p* value (-log_10_ transformed) derived from a Student's *t* test across the three biological replicates for each protein, compared with the biological replicates of a negative control protein PRO-1. The relative fold change is shown on the *x* axis using the log_2_ SILAC ratio L/H (starved/fed). The mean number of unique peptides on a log_2_ scale is represented by the spot diameter, with larger size indicating more unique peptides. Proteins with a *p* < .05 are shown in red and those with a *p* < .01 are shown in green. GO term analysis of enriched biological processes within the protein groups whose abundance either increased (*B*) or decreased (*C*) after starvation for 16 h. A threshold of more than twofold change and a *p* < .01 were used and GO term analysis was performed using the DAVID database (29). These data were plotted using the REVIGO suite (30). The *x* and *y* axes indicate semantic space used to group GO terms of related biological processes. The color and size of each bubble indicates the EASE score (log_10_ transformed) for each GO term *versus* the whole *C. elegans* proteome. The size increases with increasing log_10_ (*p* value) and the color changes from red to blue with increasing log_10_ (*p* value). Bubbles that are in close proximity indicate related GO terms.

Based on the results of the time course experiment above, we chose to focus on the 16 h of starvation time point for a subsequent in depth analysis of changes in the *C. elegans* proteome that arise in response to starvation stress. This follow-on analysis used the same treatment conditions as per the 16 h starvation time point performed in the preceding time course experiment but now included three biological replicates and protein-level fractionation. We analyzed ∼45,000 unique peptides per biological replicate, yielding >5,200 protein groups that could be quantified in at least two out of the three replicates (Supplemental Table 3 for protein-level quantitative data and Supplemental Table 4 for protein-level sequence coverage data and Supplemental Table 2 for peptide-level data). These data were used to perform a Student's *t* test across the biological replicates, comparing the SILAC ratios for each protein group with the SILAC ratios for a control protein that was widely expressed and did not change in abundance after starvation stress. These data revealed >600 protein groups that were up-regulated and >490 down-regulated in response to 16 h of starvation, using a *p* value cut-off of *p* < .05 (Fig. 2*A* red and green dots, Supplemental Table 3). The results are consistent with the proteins detected to change in abundance at 16 h post starvation in the starvation time-course experiment, demonstrating that the effects on the proteome are reproducible.

##### Analysis of the Major Biological Processes Affected by Acute Starvation

We analyzed the subset of proteins fulfilling the stringent threshold of at least a twofold change in abundance at 16 h of starvation and a *p* value of *p* < .01 to assess the regulation of distinct biological processes, as defined using GO term annotations (Fig. 2*A*, green dots, Supplemental Table 3). The GO terms indicated are those that are significantly enriched in either the up-regulated (Fig. 2*B*) or down-regulated (Fig. 2*C*) protein subsets. The significance of GO term enrichment, as reflected by the associated EASE score, is also indicated by coloration (29). Red indicates a lower significance level, while blue indicates higher significance. Up-regulated GO terms include “regulation of locomotion,” “feeding behavior,” “muscle cell differentiation,” “determination of adult lifespan,” and “chromatin organization”. The down-regulated GO terms include “cell cycle,” “DNA replication,” “lipid biosynthetic process,” “positive regulation of growth rate,” “regulation of meiosis,” “genitalia development,” and “embryo development in birth or egg hatching.” The observed reduction in abundance of proteins associated with the GO term “cell cycle” and with other growth-related GO terms was to be expected, given that germ cell proliferation is decreased after starvation (4).

**Fig. 3.**
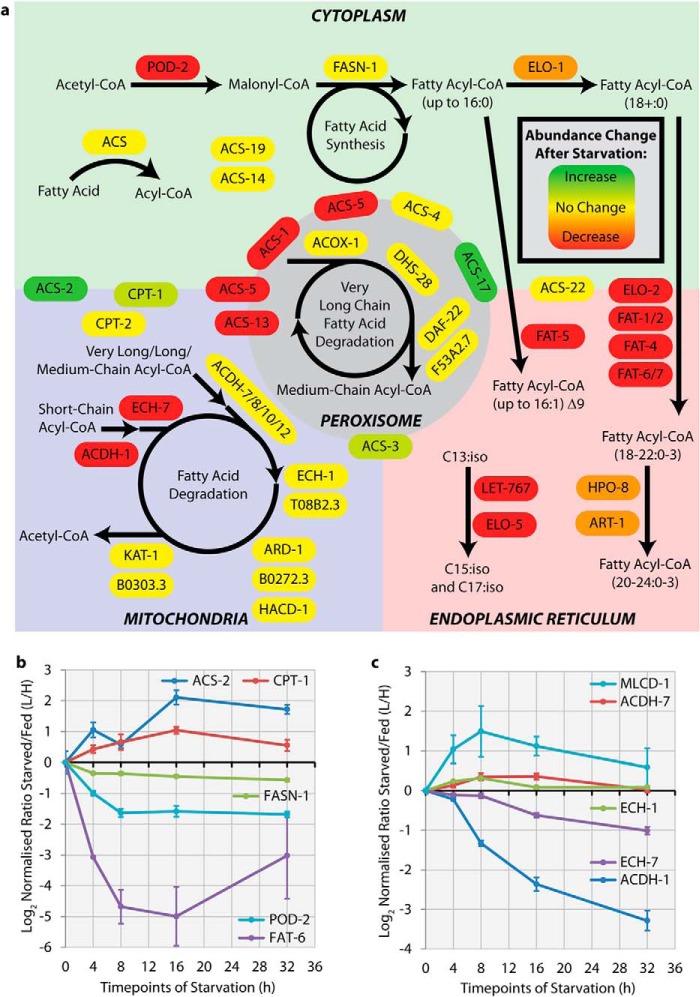
**Analysis of lipid metabolism pathways after starvation stress.** (*A*) The subcellular localization of proteins involved in lipid metabolism is indicated based upon a combination of previous publications on *C. elegans* lipid metabolism (45, 46), localization prediction tools, and homology to proteins of known location in other species. Cytoplasmic proteins are on the green background, mitochondrial proteins are on the blue background, endoplasmic reticulum proteins are on the pink background, and peroxisomal proteins are on the gray background. Proteins shown on the border either mediate the movement of metabolites between subcellular compartments or could reside in multiple locations. The color of each protein block indicates the response of the protein to 16 h of starvation, with green indicating an increase in abundance and red indicating a decrease in abundance after starvation stress. Pathways are indicated by black arrows, with circles indicating that the pathway can process the same substrate iteratively. (*B*) Line graphs showing the mean peptide SILAC ratio *versus* time after starvation stress, for key cytoplasmic, endoplasmic reticulum, and mitochondrial proteins involved in either the fatty acid synthesis or degradation pathways. Error bars show the standard error of the mean of the peptide SILAC ratio. (*C*) Line graphs showing the mean peptide SILAC ratio *versus* time after starvation stress, for key mitochondrial proteins involved in fatty acid degradation. Error bars show the standard error of the mean of the peptide SILAC ratio.

Lipids are needed for energy storage to allow organisms to cope with limited nutrient availability. In addition, they are core components of membranes and serve signaling functions. As an example of the value of our dataset for evaluating the response of whole pathways, we provide a global overview of the response of lipid metabolism to starvation stress in terms of changes in the levels of enzymes involved in the control of lipid synthesis and breakdown and grouped by subcellular localization (Fig. 3*A*, Supplemental Table 3). The key enzyme at the entry point for fatty acid synthesis is acetyl CoA carboxylase (POD-2) (Fig. 3*A*, Supplemental Table 3). This enzyme utilizes acetyl-CoA to generate malonyl-CoA, which serves as an intermediate for the fatty acid synthase (FASN-1) responsible for stepwise assembly of fatty acids up to a length of 16 carbons. Our dataset indicates that the abundance of the POD-2 protein is down-regulated by ∼threefold (Figs. 3*A* and 3*B*, supplemental Table 3). The fatty acid desaturases FAT-5, FAT1/2, FAT-4, and FAT-6/7, all of which are located in the endoplasmic reticulum, are all strongly decreased in abundance post-starvation; for example, FAT-6 levels are reduced up to ∼30 fold (Figs. 3*A* and 3*B*, Supplemental Table 3). These data further show that other enzymes generally localized in the endoplasmic reticulum, including the ELO-2, HPO-8, and ART-1 elongases, which are all required for elongation of fatty acids, are also decreased in abundance (Fig. 3*A*, Supplemental Table 3). In general, we observed that the abundance of enzymes responsible for the synthesis of fatty acids of all chain lengths and of enzymes required for desaturation decrease upon starvation.

Fatty acid degradation by beta-oxidation is required for energy production from lipids. This generally occurs in mitochondria, but very long chain fatty acids are also degraded in the peroxisome. We observe that the abundance of enzymes required for the translocation of fatty acids from the cytoplasm into mitochondria, such as acyl-CoA-synthase (ACS2) and carnitine-palmitoyl transferase (CPT-1), is increased post starvation (Figs. 3*A* and 3*B*, Supplemental Table 3). These data show that the four enzymes required for the beta-oxidation pathway of very long, long, and medium chain acyl-CoA substrates, *i.e.* acyl-CoA dehydrogenases (ACDH-7/8/10/12), Enoyl-CoA hydratase-1 (ECH-1), beta-ketothiolase (F53A2.7) and 3-ketoacyl-coA thiolase (KAT-1), do not change their abundance in response to starvation (Fig. 3*C*). These results imply that fatty acid catabolism is likely enhanced primarily by increased transport into mitochondria. In contrast, the first two enzymes in the short chain pathway of CoA beta-oxidation, *i.e.* acyl-CoA dehydrogenease (ACDH-1) and the Enoyl-CoA hydratase-7 (ECH-7), were both strongly decreased in abundance. This indicates a strong preference for the beta-oxidation of medium and long chain fatty acids for energy production rather than short chain fatty acids.

##### The Abundance of Histone H3 Variants Is Affected by Acute Starvation

**Fig. 4.**
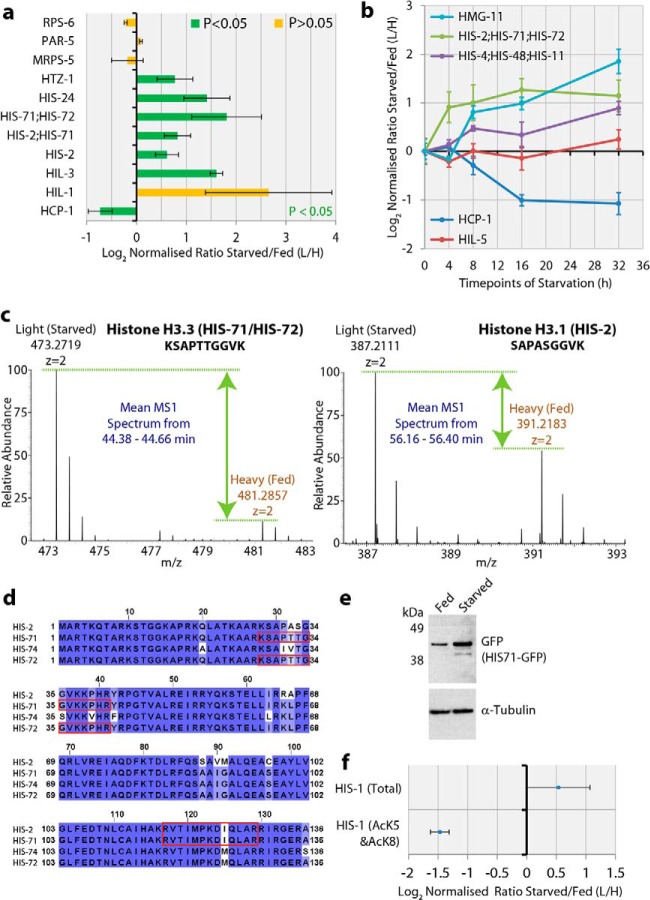
**Chromatin changes induced by starvation stress.** (*A*) Bar graph showing the response to 16 h of starvation stress for either selected chromatin-associated proteins, or negative control proteins (RPS-6, PAR-5, MRPS-5). The *y* axis indicates each protein. The bar is colored green to indicate whether the *p* value, derived from a Student's *t* test across the three biological replicates for each protein, was *p* < .05. The relative fold change is shown on the *x* axis using the log_2_ ratio L/H (starved/FED), with error bars indicating standard deviation (*n* = 3). (*B*) Line graphs showing the mean peptide SILAC ratio *versus* time after starvation stress, for selected chromatin-associated proteins. Error bars show standard error of the mean for the peptide SILAC ratio. (*C*) Mass spectra showing precursor intact peptide ions for representative SILAC pairs from peptides detected in the 16 h triplicate starvation experiment as shown in part (*A*), which are specific for either histone H3.3 (*left*), or H3.1 (*right*). (*D*) An alignment of the highly similar histone-H3-related proteins (HIS-2/71/72/74). The conservation of each sequence motif is indicated by the shade of the blue colored overlay. Red boxes surrounding the peptide sequence indicate the peptides detected in our starvation experiments. (*E*) Immunoblotting of total *C. elegans* lysates from nematodes either starved, or fed, for 16 h. The strain of *C. elegans* used contained a stable transgene with *his-71::GFP* to express a GFP-tagged histone H3.3 type-1 (HIS-71) protein. Immunoblotting was performed using either an anti-GFP antibody to detect HIS-71-GFP or an antibody specific for alpha-tubulin as a loading control. (*F*) Plot showing the response to 16 h of starvation stress for either total histone H4 (HIS-1) protein, or the double modified histone H4 peptide containing both acetylated lysine 5 and acetylated lysine 8. The relative fold change is shown on the *x* axis using the log_2_ ratio L/H (starved/fed), with error bars indicating standard deviations (*n* = 3).

Starvation can potentially trigger global changes in the chromatin landscape with major consequences for the regulation of gene expression. We observed a number of histone variants and high mobility group (HMG) chromatin proteins increased in abundance at 16 h post starvation (Figs. 4*A* and 4*B*). Starvation-induced histone variants detected here include the *C. elegans* Htz-1 H2A.Z variant, which is associated with transcriptional activation and which has been shown to act together with the PHA-4 master transcriptional regulator to drive the organogenesis of the *C. elegans* pharynx (33, 34). Furthermore, three H1 linker histones (*i.e.* HIS-24, HIL-1, and HIL-3), were also increased in abundance post starvation. H3.3 histone variants differ by only four amino acids from the canonical H3.1 protein (Fig. 4*D*), the latter being loaded during DNA replication (35). Our dataset shows that peptides specific for the core H3.1 histone (HIS-2) are increased in abundance after starvation. We also observed an increased abundance after starvation of peptides shared between H3.1 (nematode HIS-2 protein) and H3.3 (nematode HIS-71/HIS-72 proteins) and a single unique peptide from H3.3 not present in H3.1 (Figs. 4*A* and 4*C*, Supplemental Fig. S2, Supplemental Table 2 and 3).

Taking advantage of available fusion constructs and deletion mutants, we next characterized the *his-71* H3.3 variant in more detail. HIS-71 has been shown to be expressed throughout *C. elegans* development, with no expression detected in primordial germ cells (35). Using a *his-71*::GFP gene fusion, we confirmed by quantitative immunoblotting that HIS-71 is induced upon starvation (Fig. 4*E*). We also confirmed the previously reported localization of this construct to nuclei using fluorescence microscopy (Supplemental Fig. S3). Furthermore, we also detected a marked decrease in the dual acetylated form of histone H4 (HIS-1) at lysines 5 and 8 (Fig. 4*F* and Supplemental Fig. S1).

##### Data Sharing via the Encyclopedia of Proteome Dynamics

**Fig. 5.**
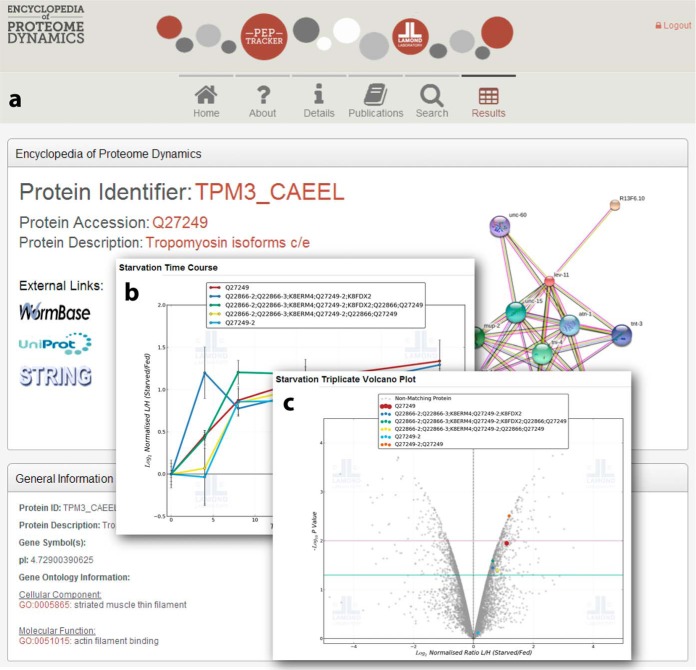
**Encyclopedia of Proteome Dynamics web-based data sharing tool.** Screen shots of data display for an example protein (tropomyosin). (*A*) Data derived from multiple databases such as Uniprot and STRING are displayed, with links to these and other external resources. (*B*) Line graphs showing the separate quantitation of tropomyosin protein groups to yield protein isoform-specific analysis, each set of protein group data is shown with a different colored line. The mean peptide SILAC ratio is plotted with error bars showing the standard error of the mean for this ratio. (*C*) Volcano plot for the 16 h starvation biological triplicate dataset. Each protein group is represented by a different colored spot and is shown in the context of all the protein groups analyzed in this experiment (colored gray). Horizontal lines indicate a *p* < .05 (green) or *p* < .01 (pink).

The Encyclopedia of Proteome Dynamics (EPD)—http://www.peptracker.com/epd/) (36)—provides a free-to-access, searchable online resource for visualizing and exploring all of the quantitative proteomics data described in this study (Fig. 5). The EPD provides convenient analysis and display of nematode protein data, with integrated links and data from Wormbase, Uniprot, and STRING for each protein. Each of these databases provides a unique resource for proteome-wide datasets and complements our protein level abundance data on the starvation response. Users can search for a protein of interest and immediately observe known interactions from STRING and basic functions associated with the protein (Fig. 5*A*). Data from this study are shown for each protein, with graphs similar to those displayed in Figs. 1*D* and 2*A*. In addition, the graphs show data derived from a combination of protein isoforms, separately to isoform-specific quantitative data. For example, tropomyosin has numerous splice isoforms that have many overlapping protein regions. This makes the analysis of such proteins more complicated. The EPD displays the quantitative information for such proteins separately, with isoform-specific and multi-isoform data being shown as different colored lines for the time-course data (Fig. 5*B*) and different colored dots for the triplicate dataset (Fig. 5*C*). For the tropomyosin protein shown in Fig. 5, this enables us to observe the quantitative differences in response to starvation between these isoforms.

## DISCUSSION

Using a combination of in-depth, quantitative proteome analyses and genetic disruption of protein function, we have characterized the global response of the *C. elegans* proteome to acute starvation. This study represents the most detailed analysis to date of protein-level responses to starvation in any organism and also provides what is currently the deepest analysis of protein expression in *C. elegans*. This analysis has revealed that starvation elicits a rapid change in protein abundance that remodels a substantial fraction of the proteome. This includes a wide range of biological processes and response mechanisms, including changes to chromatin-associated proteins. This reveals a potential mechanism for starvation to generate epigenetic effects on protein abundance and function.

The depth of our dataset provides an overview of the response within entire pathways such as lipid metabolism enzymes, which are critical to the starvation stress response. Collectively, these changes to lipid metabolism after starvation allow the organism to quickly shift from an anabolic to catabolic state, with a prioritization for mitochondrial ATP production through beta-oxidation of medium and long chain fatty acids. This dataset enables similar analyses for a plethora of pathways from metabolism to protein synthesis. To provide the community with convenient access to our data, we have deposited these data in an expanded version of the Encyclopedia of Proteome Dynamics, a searchable Web-based resource designed for optimal presentation of high-throughput proteome-wide datasets. We have also deposited all of the RAW MS data files analyzed in this study into the ProteomeXchange Consortium with the dataset identifier PXD001723.

### 

#### 

##### Chromatin Response to Starvation

A striking observation in this study was that a number of chromatin-associated proteins, including linker histones, high mobility group proteins, and histone H3.3, rapidly increased in abundance after starvation. To the best of our knowledge, this is the first report of acute starvation of a model organism regulating the abundance of chromatin-associated proteins. Interestingly, a recent proteome analysis of aging in *C. elegans* showed that histone H3.3 (HIS-71) protein abundance was ∼ threefold higher in old (5 days) *versus* young adult nematodes (37). Our own preliminary experiments indicate that the HIS-71 H3.3 variant is indeed important for survival following starvation and is also needed for nematodes to achieve a normal lifespan (data not shown). Together, these data indicate it will be important to analyze the role of HIS-71 and other chromatin-associated proteins in more detail in future studies.

Histone variants, unlike canonical histones that predominantly function to facilitate DNA-packaging, have roles in diverse processes, including chromosome segregation, DNA repair, and recombination and transcriptional silencing and activation (38). Alterations in these chromatin proteins have the potential to modulate chromatin structure and thus to affect transcription rates and cause large-scale changes in gene transcription across the genome. Our finding that acute starvation results in decreased acetylation of H4 at lysine residues K5 and K8, has profound implications for potential mechanisms that could allow food deprivation to elicit epigenetic changes in gene expression with possible long-term consequences. These histone acetylations have previously been associated with transcriptional activation and act as an epigenetic mark to allow for rapid transcriptional activation after mitosis (39). A recent study showed that starvation stress can influence subsequent generations through a small RNA-mediated mechanism (40). Our present findings suggest that the nematode may provide a useful model system to dissect mechanisms linking nutritional status with downstream epigenetic effects on gene expression and viability.

##### Metabolic Pathway Responses to Starvation

This analysis highlighted the dramatic abundance changes occurring to lipid metabolic enzymes after acute starvation stress. This showed the remodeling of these pathways to minimize energy expenditure and maximize energy production. Another aspect to this response is the need to minimize further stress to the animal, such as from reactive oxygen species (ROS) and infections. An interesting pathway that may be exploited by nematodes to achieve protection from ROS production is the up-regulation of the glyoxylate pathway after starvation. The glyoxylate pathway exists in plants, bacteria, fungi, and nematodes and converts acetyl-CoA to succinate (a substrate of complex II in the mitochondrial electron transport chain) via a by-pass of the tricarboxylic acid cycle, involving the multifunctional enzyme GEI-7/ICL-1. Use of this pathway yields less NADH (a substrate of complex I in the mitochondrial electron transport chain) per cycle. Complex II has been reported to function with less electron leakage and superoxide production when compared with complex I (41). This may explain why we observed a marked increase in ICL-1 abundance after only 4 h of starvation stress. Because, at the expense of energy production, there is a survival advantage to reduce ROS levels. Intriguingly, ICL-1 protein has been shown to be up-regulated in insulin receptor (*daf-2 null*) mutant nematodes, and this up-regulation depends on the FOXO transcription factor DAF-16; also the longevity phenotype of *daf-2* null mutants is partially suppressed by ICL-1 depletion (42). The up-regulation of ICL-1 protein we observe in this study may also contribute to the enhanced longevity of transiently starved nematodes (43).

Few studies to date have used large-scale, quantitative protein measurements to analyze the acute starvation response. One previous report on caloric restriction (not acute starvation) of nematodes identified a number of proteins that change upon calorie reduction with limited proteome resolution (44). The regulation of several proteins characterized in that study, where calorie restriction was conferred by the *eat-2* mutation, is in agreement with our present data. In addition, previous studies on the effect of starvation on proteins in mice and flies, albeit only encompassing analysis of either a small proportion of the proteome or else focused on specific pathways through targeted-MS approaches (14–16), overall are consistent with our data indicating that acute starvation inhibits fatty acid synthesis and increases beta-oxidation of fatty acids in mitochondria. In contrast, previous studies that have measured mRNA abundance changes resulting from starvation in nematodes show many differences with respect to the reported effects of starvation stress on gene expression (45, 46). These differences, which affect the conclusions as to how genes respond to starvation, were particularly evident, for example, in the response of genes encoding enzymes involved in lipid metabolism (Fig. 3). Thus, for both acetyl-CoA carboxylase (POD-2) and the majority of the acyl-CoA desaturases (FAT genes), which are involved in fatty acid biosynthesis pathways, the reported changes in mRNA abundance levels was very different from our findings regarding changes in protein abundance poststarvation. Given our current data on the effect of starvation on the proteome, combined with the fact that proteins are likely to be both the major mediators and targets of most regulatory and signaling pathways involved in the response to starvation stress, we propose that it is important in future to focus on directly evaluating changes in protein abundance, PTMs, and other protein properties in preference to exclusively measuring mRNA levels.

In summary, this study in *C. elegans* provides the first comprehensive analysis of the starvation response at a proteome-wide level, documenting changes in both protein abundance levels and PTMs. The large-scale dataset described here provides a valuable resource for future studies, both in the nematode system and in other model organisms, including humans. It will be very interesting to determine which of the effects detected in nematodes are conserved in evolution, both in terms of the targets regulated by acute starvation and the signaling mechanisms involved in mediating these responses. We envision that with the continuing improvement of proteomics instrumentation and data analysis technologies, such as global analyses of proteome-level responses during development, aging, and in response to environmental stress will increasingly provide a valuable resource for biologists to understand the control of fundamental biological responses. Such studies can also be extended in future to include additional proteomic dimensions, such as the parallel analysis of changes in the subcellular localization of the proteome, changes in protein complexes, and more comprehensive analyses of a wide range of PTMs. An important future goal will be to ensure that such data provide maximum impact to the research community through the development of effective resources for open data sharing, as shown here with the Encyclopedia of Proteome Dynamics.

## Footnotes

^1^ The abbreviations used are:

TOR: Target of Rapamycin
AMPK: 5′ adenosine monophosphate-activated protein kinase
IGF: Insulin Growth Factor
PHA: (belonging to) Pharynx
FOXA: Forkhead box A
DAF: Dauer Forming
AMP: 5′ adenosine monophosphate
AP-1: Activator Protein-1
NGM-N: Nematode Growth Medium, Nitrogen free
IPTG: Isopropyl β-d-1-thiogalactopyranoside
EZQ: Track name of a protein quantification kit produced by Life Technologies a brand under Thermo Fisher Scientific
CBQCA: Protein quantification kit produced by Life Technologies a brand under Thermo Fisher Scientific
RAW: Xcalibur Raw is a proprietary file format used by Thermo Fisher Scientific as an output for the data generated by the mass spectrometers
PRIDE: Proteomics Identification Database
GO: Gene Ontology: a database and web based resource to access a set of structured and controlled vocabularies for the community to use in annotating genes, gene products and sequences. http://www.ncbi.nlm.nih.gov/pmc/articles/PMC2238979/, http://geneontology.org/
EASE: the Expression Analysis Systematic Explorer is a customizable software application for rapid biological interpretation of gene lists that result from the analysis of microarray, proteomics, SAGE and other high-throughput genomic data. http://www.ncbi.nlm.nih.gov/pmc/articles/PMC328459/
STRING: Search Tool for the Retrieval of Interacting Genes/Proteins is a database and web interface of known and predicted protein-protein associations, integrated and transferred across organisms. http://www.ncbi.nlm.nih.gov/pmc/articles/PMC539959/, http://string-db.org/

## References

[1] Felix M. A. Braendle C. (2010) The natural history of *Caenorhabditis elegans*. Curr. Biol. 20, R965–R969 21093785 10.1016/j.cub.2010.09.050

[2] Baugh L. R. (2013) To grow or not to grow: nutritional control of development during *Caenorhabditis elegans* L1 arrest. Genetics 194, 539–555 23824969 10.1534/genetics.113.150847 PMC3697962

[3] Angelo G. Van Gilst M. R. (2009) Starvation protects germline stem cells and extends reproductive longevity in *C. elegans*. Science 326, 954–958 19713489 10.1126/science.1178343

[4] Seidel H. S. Kimble J. (2011) The oogenic germline starvation response in *C. elegans*. PloS One 6, e28074 22164230 10.1371/journal.pone.0028074 PMC3229504

[5] Sheaffer K. L. Updike D. L. Mango S. E. (2008) The Target of Rapamycin pathway antagonizes pha-4/FoxA to control development and aging. Curr. Biol. 18, 1355–1364 18804378 10.1016/j.cub.2008.07.097 PMC2615410

[6] Kenyon C. J. (2010) The genetics of ageing. Nature 464, 504–512 20336132 10.1038/nature08980

[7] Dong M. Q. Venable J. D. Au N. Xu T. Park S. K. Cociorva D. Johnson J. R. Dillin A. Yates J. R. (2007) Quantitative mass spectrometry identifies insulin signaling targets in *C. elegans*. Science 317, 660–663 17673661 10.1126/science.1139952

[8] Narbonne P. Roy R. (2009) Caenorhabditis elegans dauers need LKB1/AMPK to ration lipid reserves and ensure long-term survival. Nature 457, 210–214 19052547 10.1038/nature07536

[9] Uno M. Honjoh S. Matsuda M. Hoshikawa H. Kishimoto S. Yamamoto T. Ebisuya M. Matsumoto K. Nishida E. (2013) A fasting-responsive signaling pathway that extends life span in *C. elegans*. Cell Rep. 3, 79–91 23352664 10.1016/j.celrep.2012.12.018

[10] Vellai T. Takacs-Vellai K. Zhang Y. Kovacs A. L. Orosz L. Muller F. (2003) Genetics: Influence of TOR kinase on lifespan in *C. elegans*. Nature 426, 620 14668850 10.1038/426620a

[11] Long X. Müller F. Avruch J. (2004) TOR action in mammalian cells and in *Caenorhabditis elegans*. Curr. Top. Microbiol. Immunol. 279, 115–138 14560955 10.1007/978-3-642-18930-2_8

[12] Henderson S. T. Bonafè M. Johnson T. E. (2006) daf-16 protects the nematode *Caenorhabditis elegans* during food deprivation. J. Gerontol. A Biol. Sci. Med. Sci. 61, 444–460 16720740 10.1093/gerona/61.5.444

[13] Cui M. Cohen M. L. Teng C. Han M. (2013) The tumor suppressor Rb critically regulates starvation-induced stress response in *C. elegans*. Curr. Biol. 23, 975–980 23664972 10.1016/j.cub.2013.04.046 PMC3728909

[14] Chang Y. C. Tang H. W. Liang S. Y. Pu T. H. Meng T. C. Khoo K. H. Chen G. C. (2013) Evaluation of *Drosophila* metabolic labeling strategies for in vivo quantitative proteomic analyses with applications to early pupa formation and amino acid starvation. J. Proteome Res. 12, 2138–2150 23517121 10.1021/pr301168x

[15] Handke B. Poernbacher I. Goetze S. Ahrens C. H. Omasits U. Marty F. Simigdala N. Meyer I. Wollscheid B. Brunner E. Hafen E. Lehner C. F. (2013) The hemolymph proteome of fed and starved *Drosophila* larvae. PloS One 8, e67208 23840627 10.1371/journal.pone.0067208 PMC3688620

[16] Sabidó E. Wu Y. Bautista L. Porstmann T. Chang C. Y. Vitek O. Stoffel M. Aebersold R. (2013) Targeted proteomics reveals strain-specific changes in the mouse insulin and central metabolic pathways after a sustained high-fat diet. Mol. Syst. Biol. 9, 681 23860498 10.1038/msb.2013.36 PMC3734509

[17] Ong S. E. Blagoev B. Kratchmarova I. Kristensen D. B. Steen H. Pandey A. Mann M. (2002) Stable isotope labeling by amino acids in cell culture, SILAC, as a simple and accurate approach to expression proteomics. Mol. Cell. Proteomics 1, 376–386 12118079 10.1074/mcp.m200025-mcp200

[18] Larance M. Bailly A. P. Pourkarimi E. Hay R. T. Buchanan G. Coulthurst S. Xirodimas D. P. Gartner A. Lamond A. I. (2011) Stable-isotope labeling with amino acids in nematodes. Nat. Methods 8, 849–851 21874007 10.1038/nmeth.1679 PMC3184259

[19] Fredens J. Engholm-Keller K. Giessing A. Pultz D. Larsen M. R. Hojrup P. Moller-Jensen J. Faergeman N. J. (2011) Quantitative proteomics by amino acid labeling in *C. elegans*. Nat. Meth. 8, 845–847 10.1038/nmeth.1675 21874006

[20] Ly T. Ahmad Y. Shlien A. Soroka D. Mills A. Emanuele M. J. Stratton M. R. Lamond A. I. (2014) A proteomic chronology of gene expression through the cell cycle in human myeloid leukemia cells. eLife. 3, e01630 24596151 10.7554/eLife.01630 PMC3936288

[21] Larance M. Lamond A. I. (2015) Multidimensional proteomics for cell biology. Nat. Rev. Mol. Cell Biol. Epub ahead of print, 10.1038/nrm3970 25857810

[22] Grun D. Kirchner M. Thierfelder N. Stoeckius M. Selbach M. Rajewsky N. (2014) Conservation of mRNA and protein expression during development of *C. elegans*. Cell Rep. 6, 565–577 24462290 10.1016/j.celrep.2014.01.001

[23] Hsin H. Kenyon C. (1999) Signals from the reproductive system regulate the lifespan of *C. elegans*. Nature 399, 362–366 10360574 10.1038/20694

[24] You Y. J. Kim J. Cobb M. Avery L. (2006) Starvation activates MAP kinase through the muscarinic acetylcholine pathway in *Caenorhabditis elegans* pharynx. Cell Metab. 3, 237–245 16581001 10.1016/j.cmet.2006.02.012 PMC3433278

[25] Pourkarimi E. Greiss S. Gartner A. (2012) Evidence that CED-9/Bcl2 and CED-4/Apaf-1 localization is not consistent with the current model for *C. elegans* apoptosis induction. Cell Death Differ. 19, 406–415 21886181 10.1038/cdd.2011.104 PMC3278724

[26] Wessel D. Flügge U. I. (1984) A method for the quantitative recovery of protein in dilute-solution in the presence of detergents and lipids. Anal. Biochem. 138, 141–143 6731838 10.1016/0003-2697(84)90782-6

[27] Ritorto M. S. Cook K. Tyagi K. Pedrioli P. G. Trost M. (2013) Hydrophilic strong anion exchange (hSAX) chromatography for highly orthogonal peptide separation of complex proteomes. J. Proteome Res. 12, 2449–2457 23294059 10.1021/pr301011r PMC3679572

[28] Cox J. Mann M. (2008) MaxQuant enables high peptide identification rates, individualized p.p.b.-range mass accuracies and proteome-wide protein quantification. Nat. Biotechnol. 26, 1367–1372 19029910 10.1038/nbt.1511

[29] Huang D. W. Sherman B. T. Lempicki R. A. (2009) Systematic and integrative analysis of large gene lists using DAVID bioinformatics resources. Nat. Protoc. 4, 44–57 19131956 10.1038/nprot.2008.211

[30] Supek F. Bošnjak M. Škunca N. Šmuc T. (2011) REVIGO Summarizes and visualizes long lists of gene ontology terms. Plos One 6, e21800 21789182 10.1371/journal.pone.0021800 PMC3138752

[31] Raharjo W. H. Logan B. C. Wen S. B. Kalb J. M. Gaudet J. (2010) In vitro and in vivo characterization of *Caenorhabditis elegans* PHA-4/FoxA response elements. Dev. Dynam. 239, 2219–2232 10.1002/dvdy.22359 20623595

[32] Murphy C. T. McCarroll S. A. Bargmann C. I. Fraser A. Kamath R. S. Ahringer J. Li H. Kenyon C. (2003) Genes that act downstream of DAF-16 to influence the lifespan of *Caenorhabditis elegans*. Nature 424, 277–283 12845331 10.1038/nature01789

[33] Updike D. L. Mango S. E. (2006) Temporal regulation of foregut development by HTZ-1/H2A.Z and PHA-4/FoxA. PLoS Genet. 2, 1500–1510 10.1371/journal.pgen.0020161 PMC1584275 17009877

[34] Whittle C. M. McClinic K. N. Ercan S. Zhang X. M. Green R. D. Kelly W. G. Lieb J. D. (2008) The genomic distribution and function of histone variant HTZ-1 during *C. elegans* embryogenesis. PLoS Genet. 4, e1000187 18787694 10.1371/journal.pgen.1000187 PMC2522285

[35] Ooi S. L. Priess J. R. Henikoff S. (2006) Histone H3.3 variant dynamics in the germline of *Caenorhabditis elegans*. PLoS Genet. 2, 883–895 10.1371/journal.pgen.0020097 PMC1484599 16846252

[36] Larance M. Ahmad Y. Kirkwood K. J. Ly T. Lamond A. I. (2013) Global subcellular characterization of protein degradation using quantitative proteomics. Mol. Cell. Proteomics 12, 638–650 23242552 10.1074/mcp.M112.024547 PMC3591657

[37] Liang V. Ullrich M. Lam H. Chew Y. L. Banister S. Song X. Zaw T. Kassiou M. Gotz J. Nicholas H. R. (2014) Altered proteostasis in aging and heat shock response in *C. elegans* revealed by analysis of the global and de novo synthesized proteome. Cell Mol. Life Sci. In press. 10.1007/s00018-014-1558-7 PMC4131143 24458371

[38] McBryant S. J. Lu X. Hansen J. C. (2010) Multifunctionality of the linker histones: An emerging role for protein-protein interactions. Cell Res. 20, 519–528 20309017 10.1038/cr.2010.35 PMC2919278

[39] Zhao R. Nakamura T. Fu Y. Lazar Z. Spector D. L. (2011) Gene bookmarking accelerates the kinetics of post-mitotic transcriptional re-activation. Nat. Cell Biol. 13, 1295–1304 21983563 10.1038/ncb2341 PMC3210065

[40] Rechavi O. Houri-Ze'evi L. Anava S. Goh W. S. Kerk S. Y. Hannon G. J. Hobert O. (2014) Starvation-induced transgenerational inheritance of small RNAs in *C. elegans*. Cell 158, 277–287 25018105 10.1016/j.cell.2014.06.020 PMC4377509

[41] Yankovskaya V. Horsefield R. Törnroth S. Luna-Chavez C. Miyoshi H. Leger C. Byrne B. Cecchini G. Iwata S. (2003) Architecture of succinate dehydrogenase and reactive oxygen species generation. Science 299, 700–704 12560550 10.1126/science.1079605

[42] Shen E. Z. Song C. Q. Lin Y. Zhang W. H. Su P. F. Liu W. Y. Zhang P. Xu J. J. Lin N. Zhan C. Wang X. Shyr Y. Cheng H. Dong M. Q. (2014) Mitoflash frequency in early adulthood predicts lifespan in *Caenorhabditis elegans*. Nature. 508, 128–132 24522532 10.1038/nature13012

[43] Kaeberlein T. L. Smith E. D. Tsuchiya M. Welton K. L. Thomas J. H. Fields S. Kennedy B. K. Kaeberlein M. (2006) Lifespan extension in *Caenorhabditis elegans* by complete removal of food. Aging Cell. 5, 487–494 17081160 10.1111/j.1474-9726.2006.00238.x

[44] Yuan Y. Kadiyala C. S. Ching T. T. Hakimi P. Saha S. Xu H. Yuan C. Mullangi V. Wang L. Fivenson E. Hanson R. W. Ewing R. Hsu A. L. Miyagi M. Feng Z. (2012) Enhanced energy metabolism contributes to the extended life span of calorie-restricted *Caenorhabditis elegans*. J. Biol. Chem. 287, 31414–31426 22810224 10.1074/jbc.M112.377275 PMC3438970

[45] Van Gilst M. R. Hadjivassiliou H. Jolly A. Yamamoto K. R. (2005) Nuclear hormone receptor NHR-49 controls fat consumption and fatty acid composition in *C. elegans*. PLoS Biol. 3, e53 15719061 10.1371/journal.pbio.0030053 PMC547972

[46] Van Gilst M. R. Hadjivassiliou H. Yamamoto K. R. (2005) A *Caenorhabditis elegans* nutrient response system partially dependent on nuclear receptor NHR-49. Proc. Natl. Acad. Sci. U.S.A. 102, 13496–13501 16157872 10.1073/pnas.0506234102 PMC1201344

